# High-temperature ethanol fermentation from pineapple waste hydrolysate and gene expression analysis of thermotolerant yeast *Saccharomyces cerevisiae*

**DOI:** 10.1038/s41598-022-18212-w

**Published:** 2022-08-17

**Authors:** Huynh Xuan Phong, Preekamol Klanrit, Ngo Thi Phuong Dung, Sudarat Thanonkeo, Mamoru Yamada, Pornthap Thanonkeo

**Affiliations:** 1 Department of Biotechnology, Faculty of Technology, Khon Kaen University, Khon Kaen, 40002 Thailand; 2 Department of Microbial Biotechnology, Biotechnology Research and Development Institute, Can Tho University, Can Tho, 900000 Vietnam; 3 Walai Rukhavej Botanical Research Institute, Mahasarakham University, Maha Sarakham, 44150 Thailand; 4 Department of Biological Chemistry, Faculty of Agriculture, Yamaguchi University, Yamaguchi, 753-8515 Japan; 5 Research Center for Thermotolerant Microbial Resources, Yamaguchi University, Yamaguchi, 753-8315 Japan; 6 Center for Alternative Energy Research and Development (AERD), Khon Kaen University, Khon Kaen, 40002 Thailand

**Keywords:** Industrial microbiology, Industrial microbiology, Molecular biology

## Abstract

High-temperature ethanol fermentation by thermotolerant yeast is considered a promising technology for ethanol production, especially in tropical and subtropical regions. In this study, optimization conditions for high-temperature ethanol fermentation of pineapple waste hydrolysate (PWH) using a newly isolated thermotolerant yeast, *Saccharomyces cerevisiae* HG1.1, and the expression of genes during ethanol fermentation at 40 °C were carried out. Three independent variables, including cell concentration, pH, and yeast extract, positively affected ethanol production from PWH at 40 °C. The optimum levels of these significant factors evaluated using response surface methodology (RSM) based on central composite design (CCD) were a cell concentration of 8.0 × 10^7^ cells/mL, a pH of 5.5, and a yeast extract concentration of 4.95 g/L, yielding a maximum ethanol concentration of 36.85 g/L and productivity of 3.07 g/L. Gene expression analysis during high-temperature ethanol fermentation using RT–qPCR revealed that the acquisition of thermotolerance ability and ethanol fermentation efficiency of *S. cerevisiae* HG1.1 are associated with genes responsible for growth and ethanol stress, oxidative stress, acetic acid stress, DNA repair, the pyruvate-to-tricarboxylic acid (TCA) pathway, and the pyruvate-to-ethanol pathway.

## Introduction

Increasing energy demands encourage scientists to find low-cost, clean, renewable, and sustainable alternative energy sources^1–3^. A comparative study of literature on various alternative fuels, such as ethanol, vegetable oils, microbial oils, biomass, glycerol, biodiesel, and hydrogen, has been reported^4,5^. Commercial ethanol for biofuel is produced from feedstocks such as sugarcane, corn, and cassava. These raw materials, which are also food for human needs and animal feed, are competitively priced^6^. Agricultural wastes, particularly lignocellulosic materials, have been considered promising for second-generation bioethanol production. Pineapple peel, core, stem, and leaves are byproducts of pineapple processing (approximately 50% (w/w) of the pineapple weight)^7^. These byproducts are highly biodegradable and rich in proteins and carbohydrates, which are promising raw and abundant materials for ethanol production^8,9^. Thailand and Vietnam are the top countries in pineapple production, producing 2.21 and 0.59 million metric tons, accounting for 8.91% and 2.38% of the world's production, respectively^10^.

In summer, the temperature in Thailand and Vietnam dramatically increases, which will increase with global warming. Furthermore, the temperature inside a bioreactor may rise from 30 °C to approximately 40 °C during ethanol fermentation^11^. High temperatures inhibit cell growth and the metabolic activity of yeast cells, resulting in a reduction in ethanol yield and volumetric ethanol productivity^12,13^. Therefore, the use of thermotolerant microorganisms is a promising approach to solving the problem of ethanol production at high temperatures. There are several advantages of using high-temperature ethanol fermentation, such as decreased costs associated with a cooling system, higher yields obtained in saccharification, and reduced risk of contamination by bacteria^14,15^. Even though many thermotolerant yeasts can tolerate and ferment at high temperatures, several stresses, e.g., thermal, ethanol, osmotic, ionic, lignocellulosic inhibitors, and reactive oxygen species (ROS), are unfavorable conditions for yeast growth and fermentation activity. Denaturation of DNA, proteins, lipids, and essential cellular structures of yeast cells under stressful situations has been previously reported^16–19^. However, the molecular mechanism conferring thermotolerance acquisition during high-temperature ethanol fermentation using PWH as feedstock has not yet been evaluated.

*S. cerevisiae* can reproduce in anaerobic and aerobic conditions and accumulate ethanol at high concentrations, making it the preferred choice for starter cultures for beverage and food fermentations^20^. Recently, *S. cerevisiae* has become one of the most engineered yeasts for ethanol production from the agricultural, kitchen, industrial, and lignocellulosic wastes^21,22^. *S. cerevisiae* HG1.1 is one of several thermotolerant yeasts isolated from soil samples in Vietnam^23^. This newly isolated yeast can grow and produce ethanol at a temperature up to 45 °C, using a YM medium containing 160 g/L glucose. Furthermore, it can tolerate ethanol and acetic acid up to 14% (v/v) and 4 g/L, respectively, when growing on YM agar at 35 °C^23^. Since its ethanol production potential using agricultural waste as feedstock has never been elucidated. Therefore, this newly isolated yeast was chosen for ethanol production under high-temperature conditions using PWH as feedstock in this study.

The disadvantages of a single variable optimization technique, for instance, missing the interactions between the experimental factors and requiring a large number of experiments, can be eliminated by a statistical experimental model such as response surface methodology (RSM) based on a central composite design (CCD)^24^. Statistical tools such as RSM are used for experimental design, determining the positive and negative variables and their interactions, predicting the optimal equation for optimization with the cost-effective process, and reducing experimental runs^25,26^. Several recent reports have used this statistical method to optimize different medium compositions for bioethanol fermentation^27–29^. This study used a statistical optimization methodology to investigate ethanol production from PWH at high temperatures by the newly isolated thermotolerant yeast *S. cerevisiae* HG1.1. In addition, reverse transcription quantitative real-time polymerase chain reaction (RT–qPCR) was applied to analyze the expression levels of selected genes responsible for growth and ethanol stress (*ATP6*, *OLE1*, *ERG8*), oxidative stress (*GLR1*, *SOD1*), DNA repair (*RAD14*, *MRE11*, *POL4*), the pyruvate-to-tricarboxylic acid (TCA) pathway (*PDA1*, *CIT1*, *LYS21*), the pyruvate-to-ethanol pathway (*PDC1*, *ADH1*, *ADH2*), and acetic acid stress (*ACS1*, *ALD2*) in *S. cerevisiae* HG1.1. This study could provide the optimum conditions for ethanol production from PWH and help better understand the molecular mechanism by which yeast cells acquire thermotolerance and fermentation efficiency during high-temperature ethanol fermentation.

## Materials and methods

### Strain and culture media

The newly isolated thermotolerant yeast *S. cerevisiae* HG1.1, isolated from soil samples from Vietnam, was used in this study. Isolation, screening, and selection of this thermotolerant yeast strain were described by Phong et al.^23^. The yeast culture was stored at the Department of Biotechnology, Faculty of Technology, Khon Kaen University, Thailand.

The medium used was YM medium (0.3% yeast extract, 0.3% malt extract, 0.5% peptone, and 1.0% D-glucose). Yeast inoculum was prepared by transferring one colony of a 24-h culture grown on a slant of YM agar to a test tube containing 10 mL of YM broth and incubating on a rotary shaker at 35 °C and 100 rpm for 18 h. Then, 10 mL of preculture was inoculated into a 500-mL Erlenmeyer flask containing 200 mL of YM broth (pH 5.0) and incubated on a rotary shaker under the same conditions for 18 h. The final yeast cell concentrations were approximately 1.0 × 10^8^–2.5 × 10^8^ cells/mL. The active yeast cells were collected by centrifugation and used as a starter culture.

### Plant material

Pineapple (*Ananas comosus* L. cv. Pattavia) wastes (pineapple peels and core) were collected in May 2018 from the Food Services Center, Khon Kaen University, Khon Kaen province, Thailand, with the permission of the Khon Kaen University Office. The plant used in this study is not wild but cultivated in Nong Khai province, Thailand. A voucher specimen (dried material) was deposited at the Department of Biotechnology, Faculty of Technology, Khon Kaen University, with the code number: KKUDB-PPC-2018-01. All methods were performed following relevant guidelines in the method section.

### PWH preparation and chemical composition analysis

Pineapple wastes were collected and chopped into small pieces. They were dried under natural conditions (sun drying for 3 days) and in a hot air oven for 24 h. The dried pineapple wastes were milled by a laboratory blender, mixed in a single lot, and stored prior to use. The fiber compositions of dried pineapple wastes were analyzed using an Ankom Fiber Analyzer^30^ at the Animal Science Laboratory, Faculty of Agriculture, Khon Kaen University.

PWH was prepared by transferring dried pineapple wastes to 0.5% (v/v) sulfuric acid (H_2_SO_4_) and heating at 121 °C for 15 min^31^. After hydrolysis, the pellet was removed by centrifugation, and the resulting supernatant was collected and kept at − 20 °C. The sugar compositions, acetic acid, formic acid, and furfural were analyzed using high-performance liquid chromatography (HPLC) at Central Laboratory, Faculty of Technology, Khon Kaen University and Central Laboratory (Thailand) Co., Ltd., Khon Kaen. Minerals, such as nitrogen, phosphorus, and magnesium, were analyzed at the Chemical Analysis Laboratory, Agricultural Development Research Center in Northeast Thailand, Khon Kaen, Thailand.

### Effect of inorganic nitrogen sources on ethanol fermentation

Inorganic nitrogen sources have been shown to affect ethanol production under high-temperature fermentation conditions. In this study, based on the literature reviews, various inorganic nitrogen sources, including urea [CO(NH_2_)_2_], ammonium sulfate [(NH_4_)_2_SO_4_], ammonium nitrate [NH_4_NO_3_], and diammonium phosphate [DAP, (NH_4_)_2_HPO_4_] at different concentrations^27,32^ were determined for their effect on ethanol production by *S. cerevisiae* HG1.1. The ethanol fermentation was conducted in triplicate using a 250-mL Erlenmeyer flask containing 100 mL of PWH (pH 5.0) supplemented with various nitrogen sources at different concentrations and an initial yeast cell concentration of 5.0 × 10^6^ cells/mL. All flasks were incubated at 40 °C on a rotary shaker at 100 rpm. Samples were withdrawn every 12 h and subjected to ethanol and total sugar analyses.

### Optimization of ethanol production at high temperature

Based on the literature reviews, several environmental factors or variables affect ethanol production under high-temperature conditions. In this study, some influence factors include initial yeast cell concentration, pH of the fermentation medium, manganese (II) sulfate (MnSO_4_·H_2_O), zinc sulfate (ZnSO_4_·7H_2_O), magnesium sulfate (MgSO_4_·7H_2_O), potassium dihydrogen phosphate (KH_2_PO_4_), and yeast extract were chosen^12,23,27,28,32,33^. The significant independent factors positively affecting ethanol production from PWH by *S. cerevisiae* HG1.1 were screened and selected using PBD. The codes and actual values of the independent factors are presented in Table 1. The batch ethanol fermentation experiments were performed in triplicate using a 250-mL Erlenmeyer flask containing 100 mL of PWH (pH 5.0). The ethanol concentration was set as the response variable in this study.

**Table 1:** Codes and actual values of the independent factors for the designed experiment.

Code	Factor	Unit	Low level (− 1)	High level (+ 1)
A	Initial cell concentration	cells/mL	1 × 10^6^	5 × 10^7^
B	pH	g/L	4.0	6.0
C	Manganese (II) sulfate (MnSO_4_·H_2_O)	g/L	0.02	1.5
D	Zinc sulfate ZnSO_4_·7H_2_O	g/L	0.02	1.5
E	Magnesium sulfate (MgSO_4_·7H_2_O)	g/L	0.05	2.0
F	Potassium dihydrogen phosphate (KH_2_PO_4_)	g/L	0.05	2.0
G	Yeast extract	g/L	0.0	6.0

The significant independent variables selected based on PBD were subjected to an optimization experiment using the RSM based on the CCD. The confirmatory experiment was carried out using the optimized conditions from the response surface analysis.

### RT–qPCR analysis of gene expression in *S. cerevisiae* HG1.1 under high-temperature ethanol fermentation

The yeast inoculum was transferred into a 250-mL Erlenmeyer flask containing 100 mL PWH (pH 5.5) supplemented with 4.95 g/L yeast extract with an initial cell concentration of 8.0 × 10^7^ cells/mL. All flasks were incubated on a rotary shaker at 100 rpm under four different fermentation conditions: (1) unstressed condition (flasks were incubated at 30 °C for 9 h); (2) heat shock condition (flasks were incubated at 30 °C for 9 h, then shifted to 40 °C for 30 min); (3) short-term heat stress (flasks were incubated at 30 °C for 9 h, then shifted to 40 °C for 3 h); and (4) long-term heat stress (flasks were incubated at 40 °C for 9 h). Yeast cells were harvested at specific time points (i.e., 9 h for unstressed, 9 h and 30 min for heat shock, 12 h for short-term heat stress, and 9 h for long-term heat stress) by centrifugation at 5,000 rpm and 4 °C for 5 min, and then subjected to total RNA isolation using an RNA extraction kit (GF-1 Total RNA extraction kit, Vivantis, USA) with some modifications as described by Techaparin et al.^34^. The RNA concentration in each sample was measured and adjusted using a BioDrop μLITE (BioDrop Ltd, UK). RT–qPCR was performed in triplicate on a 7500 Fast Real-Time PCR System using the qPCRBIO SyGreen One-Step Detect Lo-ROX (PCR Biosystems, London, UK). The reactions were conducted with a total volume of 20 μL containing 1 μL RNA sample (100 ng RNA), 0.8 μL of each specific forward and reverse primer, 1 μL 20 × RTase, 10 μL 2 × qPCRBIO Sygreen One-Step mix, and 6.4 μL RNase-free water. The thermal cycling conditions were as follows: 45 °C for 30 min; 95 °C for 2 min; 40 cycles of 95 °C for 15 s, and 60 °C for 1 min. A list of primer pairs used for RT–qPCR is shown in Table 2. The RNase-free water was used instead of the RNA template for the negative control. The actin gene (*ACT1*) was used as an internal control. The relative gene expression was calculated using the 2^−ΔΔCT^ method in which the target gene amount was adjusted to the reference gene (*ACT1* gene).

**Table 2:** A list of primer pairs used for RT−qPCR.

No	Gene	Primer	Nucleotide sequence (5′–3′)
1	*ATP6*	ATP6–F	GAGATTAGACTATTATTTGG
		ATP6–R	TACTAATGGTAATGGTGTAC
2	*OLE1*	OLE1–F	CCAGCAGTGGCATTGTCGAC
		OLE1–R	CCCTTCAACGGAAGCACAACC
3	*ERG8*	ERG8–F	GTGCCCCAGGGAAAGCGTTA
		ERG8–R	ACTGTGACTAAACCTGCCGA
4	*GLR1*	GLR1–F	AGAGCTGCATCTTATGGTGC
		GLR1–R	CCAATATAACCAGCGCCAAC
5	*SOD1*	SOD1–F	TAAAGGGTGATGCCGGTGTCT
		SOD1–R	TTAGACCAATGACACCACAGG
6	*RAD14*	RAD14–F	GAGTACGATTTTGCCACCATGC
		RAD14–R	CCTTCTTCTCCACCCCATTT
7	*MRE11*	MRE11–F	TGTTGCATGGGTGACAAGCC
		MRE11–R	GACCCCATATCACCATATCCAG
8	*POL4*	POL4–F	CAGTTGCATCACAAAGTGGG
		POL4–R	GCAATTTCCGACCCAATGCCG
9	*LYS21*	LYS21–F	TCTCCCGTAGCATCCGAACA
		LYS21–R	CCACCTTCCAAAGCAGTGTA
10	*PDA1*	PDA1–F	CATCAGAAGAATGGAGATGGC
		PDA1–R	GGTACCCATACCGTACTTGTTG
11	*CIT1*	CIT1–F	GGGAAGGTTCCGTGTTAGAC
		CIT1–R	CGTTACCACCTTCATGATCAG
12	*PDC1*	PDC1–F	AGATGGGCTGGTAACGCCAA
		PDC1–R	GCATCAGCCAAGATAACTGGG
13	*ADH1*	ADH1–F	CTCTGGTGTCTGTCACACTG
		ADH1–R	CTGTAACCCATAGCCTTGGC
14	*ADH2*	ADH2–F	CCCAGTTCCAAAGCCAAAGCC
		ADH2–R	CTGTAACCCATCGCCTTAGC
15	*ACS1*	ACS1–F	GCATGGTTCCTCAACGGCCA
		ACS1–R	GGATCCTCAGAATCAACGGG
16	*ALD2*	ALD2–F	TCGAAACTGTGAACCCAGCTAC
		ALD2–R	ATAACCGTGTTACCGGCTGC
17	*ACT1*	ACT1–F	GGTAGACCAAGACACCAAGG
		ACT1–R	GAAGTCCAAGGCGACGTAAC

### Analytical methods and data analysis

Viable cell concentration was determined by a haemacytometer using the methylene blue staining technique^35^. The total sugars were analyzed by the phenol sulfuric acid method^36^ using a spectrophotometer (UV-1601, Shimadzu). The ethanol concentration was determined by gas chromatography (GC-14B, Shimadzu) using a packed column of polyethylene glycol (PEG-20 M) with a flame ionization detector^37^. The following equations were used to calculate the fermentation parameters: ethanol yield (Y_p/s_, g/g) = P_E_/[S_0_ − S_t_]; volumetric ethanol productivity (Q_p_, g/L.h) = P_E_/t; yield efficiency (E_y_, %) = [Y_p/s_/0.511] × 100; sugar consumption (S_c_, %) = [S_t_/S_0_] × 100, where P_E_ is ethanol concentration (g/L), S_0_ is initial sugar concentration (g), S_t_ is sugar concentration (g) at time t, and t is fermentation time (h). The data are expressed as the mean ± standard deviation (SD). Analysis of variance was used to evaluate the differences among the treatments using Duncan's multiple range tests (DMRT). The statistical analysis was carried out using Statgraphics Centurion XV (Statpoint Technologies Inc., USA).

## Results and discussion

### Composition of dried pineapple waste and PWH

The dried pineapple waste had high contents of hemicelluloses (28.81%) and cellulose (16.57%), and the total crude fiber was 48.72%, while lignin comprised only 3.04% of the total dry matter. Previous studies by Niwaswong et al.^38^ reported that raw pineapple peel comprised 9.43% hemicellulose, 20.44% cellulose, and 41.21% lignin. Choonut et al.^8^ showed that 51.13% hemicellulose, 37.68% cellulose, and 10.24% lignin were detected in pineapple peel after hot water pretreatment at 100 °C for 240 min. The cellulose content of pineapple waste used in this study was lower than that of other agricultural wastes, such as rice straw (32–47% cellulose)^39^ and corn stover (38–40% cellulose)^40^. However, the cellulose and hemicellulose contents were greater than those of yam peel (5.7% cellulose, 5.1% hemicellulose) and cassava peel (12.7% cellulose, 5.5% hemicellulose)^41^.

Glucose and fructose were the principal sugars found in PWH, accounting for 41.11 and 40.87 g/L, respectively, while sucrose and maltose were not detected. Xylose and arabinose were also detected at 5.34 and 4.42 g/L, respectively. Rattanapoltee and Kaewkannetra^31^ reported that only 18.41 g/L glucose and 24.55 g/L fructose were present in pineapple peel hydrolysate. The total sugar concentration was only 55.91 g/L. Niwaswong et al.^38^ reported 82.10 g/L reducing sugars by dilute acid hydrolysis of pineapple peel waste. Formic acid, acetic acid, and furfural are considered inhibitors that are derived from acid hydrolysis. The acetic acid concentration of PWH was 8.39 g/L, whereas the concentrations of formic acid and furfural were 0.96 g/L and 0.36 mg/L, respectively, which were lower than those reported by Rattanapoltee and Kaewkannetra^31^.

The total sugars of PWH were 103.03 g/L, which was as high as the total sugars found in orange peel hydrolysate (101 g/L)^33^ and banana peel hydrolysate (155 g/L)^42^. Furthermore, the PWH also contains some minerals, such as nitrogen (686 mg/L), phosphorus (274 mg/L), magnesium (126 mg/L), manganese (34 mg/L), and zinc (5 mg/L), which are essential for yeast growth and metabolic activity. Based on the sugar and mineral contents presented in PWH, it was considered a promising potential feedstock for ethanol and other biochemical production.

### Effect of inorganic nitrogen sources on ethanol production

The supplementation of inorganic nitrogen sources into the PWH did not significantly increase the final ethanol concentrations compared to the control treatment without inorganic nitrogen supplementation. The highest ethanol concentrations of 34.60, 34.56, and 33.55 g/L were achieved from the medium supplemented with NH_4_NO_3_, (NH_4_)_2_SO_4_, and (NH_4_)_2_HPO_4_, respectively, which were not significantly different from the control treatment (33.54 g/L). Furthermore, supplementation with CO(NH_2_)_2_ resulted in a lower ethanol concentration than the control (Table 3). Due to the low sugar content of PWH, *S. cerevisiae* HG1.1 quickly converted all sugars to reach the maximum ethanol concentration with a low consumption level of nitrogen sources. On the other hand, PWH may contain sufficient nitrogen sources for yeast growth and metabolic activity. Generally, nitrogen is essential when the fermentation process is carried out at a high initial sugar concentration (for example, greater than 200 g/L)^43^. The results in the present study coincide with those reported by Charoensopharat et al.^32^ and Arora et al.^44^.

**Table 3:** Kinetic parameters of ethanol production from PWH supplemented with various inorganic nitrogen sources and concentrations at 40 °C for 18 h.

Concentration (g/L)	Parameter (Mean ± SD)			
	P_E_ (g/L)	Y_p/s_ (g/g)	E_y_ (%)	S_c_ (%)
0	33.45 ± 0.78^abc^	0.43 ± 0.00	81.57 ± 1.53	76.94 ± 1.61
*Ammonium nitrate (AN)*				
0.25	34.60 ± 0.49^a^	0.48 ± 0.01	92.30 ± 2.17	71.47 ± 1.54
0.50	34.27 ± 0.17^ab^	0.49 ± 0.01	96.08 ± 1.24	70.34 ± 1.14
0.75	33.56 ± 0.12^abc^	0.44 ± 0.01	86.98 ± 1.36	75.88 ± 1.13
1.0	33.99 ± 0.14^ab^	0.46 ± 0.01	90.29 ± 1.17	72.62 ± 1.00
*Ammonium sulfate (AS)*				
0.25	34.56 ± 0.24^a^	0.46 ± 0.03	90.34 ± 2.47	73.79 ± 1.97
0.50	33.92 ± 0.27^ab^	0.45 ± 0.01	87.71 ± 1.20	76.28 ± 1.21
0.75	33.90 ± 0.44^ab^	0.47 ± 0.03	92.21 ± 2.41	72.46 ± 1.34
1.0	33.93 ± 0.63^ab^	0.45 ± 0.02	87.34 ± 1.73	75.02 ± 1.02
*Diammonium phosphate (DAP)*				
0.25	29.56 ± 0.21^ g^	0.43 ± 0.02	84.86 ± 1.63	67.22 ± 1.25
0.50	32.53 ± 0.98^ cd^	0.43 ± 0.01	83.75 ± 2.42	76.60 ± 1.00
0.75	33.55 ± 0.71^abc^	0.45 ± 0.02	88.73 ± 3.04	74.33 ± 1.44
1.0	33.11 ± 0.88^bcd^	0.44 ± 0.01	87.01 ± 1.49	73.43 ± 0.75
*Urea (UR)*				
0.25	29.80 ± 0.48^ fg^	0.42 ± 0.00	81.64 ± 0.27	70.33 ± 0.90
0.50	31.06 ± 0.82^ef^	0.45 ± 0.02	87.60 ± 1.79	69.92 ± 0.16
0.75	31.82 ± 0.47^de^	0.43 ± 0.02	84.91 ± 1.22	73.77 ± 1.66
1.0	31.94 ± 0.25^de^	0.43 ± 0.01	84.03 ± 0.91	73.39 ± 1.04

P_E_: ethanol concentration (g/L); Y_p/s_: ethanol yield (g/g); E_y_: yield efficiency (%); and S_c_: sugar consumption (%). Mean values with different letters in the same column are significantly different (*p* value < 0.05) based on DMRT analysis.

The C/N ratio of the fermentation medium also played a crucial role in ethanol production. One study reported the optimum C/N ratio of 7.9 for ethanol production from sago starch using recombinant *S. cerevisiae* YKU 131^45^, while the other reported the value of 35.2 for ethanol production from tapioca starch using co-culture of *Aspergillus niger* and *S. cerevisiae*^46^. In this study, the C/N ratio of the fermentation medium was not determined; thus, further study is needed to clarify this hypothesis. Based on the results in this study, inorganic nitrogen was not selected as the independent variable in the experiment on screening factors that affected ethanol production using a Plackett–Burman design (PBD).

### Screening of significant factors for ethanol production by *S. cerevisiae* HG1.1 using a Plackett and Burman design (PBD)

**Figure 1:**
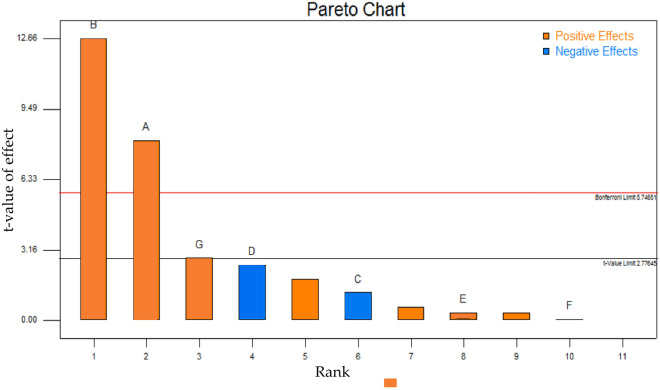
Pareto chart of standardized effects for the Plackett–Burman design of initial cell concentration (**A**), initial pH (**B**), MnSO_4_.H_2_O (**C**), ZnSO_4_.7H_2_O (**D**), MgSO_4_.7H_2_O (**E**), KH_2_PO_4_ (**F**), and yeast extract (**G**).

The PBD used 7 independent factors and 12 experimental runs. The maximal ethanol concentration of 32.73 g/L and volumetric ethanol productivity of 2.18 g/L.h were achieved after 15 h of fermentation at 40 °C (Table 4). Three factors, including initial cell concentration (A), pH (B), and yeast extract (G), were the most significant variables in ethanol production from PWH by the thermotolerant yeast *S. cerevisiae* HG1.1, with *p* values < 0.05 (Table 5). Analysis of the adequate levels of these three crucial factors showed that the most influential factor was pH (*p* value was 0.0002), and yeast extract was the most negligible influential factor (*p* value was 0.0485). The selected model was significant (*p* value < 0.005), with a high confidence level based on the values of R-squared (0.9837) and adjusted R-squared (0.9551). Based on the *t* value limit on the Pareto chart (Fig. 1), three variables, including initial cell concentration (A), pH (B), and yeast extract (G), were considered significant variables. All three variables positively affected ethanol production from PWH using *S. cerevisiae* HG1.1.

**Table 4:** Plackett–Burman design matrix for the screening of variable effects on ethanol production from PWH using *S. cerevisiae* HG1.1.

Std	Run	Variables							Responses	
		A	B	C	D	E	F	G	Ethanol (g/L)	Productivity (g/L.h)
5	1	1.0 × 10^6^	4.0	1.50	0.02	2.00	2.00	0	0.93 ± 0.15	0.06 ± 0.01
6	2	1.0 × 10^6^	4.0	0.02	1.50	0.05	2.00	9	2.18 ± 0.71	0.15 ± 0.05
12	3	1.0 × 10^6^	4.0	0.02	0.02	0.05	0.05	0	0.81 ± 0.11	0.05 ± 0.01
10	4	1.0 × 10^6^	6.0	1.50	1.50	0.05	0.05	0	11.95 ± 1.02	0.80 ± 0.07
11	5	5.0 × 10^7^	4.0	1.50	1.50	2.00	0.05	0	6.15 ± 0.25	0.41 ± 0.02
7	6	5.0 × 10^7^	4.0	0.02	0.02	2.00	0.05	9	15.04 ± 1.76	1.00 ± 0.12
1	7	5.0 × 10^7^	6.0	0.02	1.50	2.00	2.00	0	27.15 ± 1.40	1.81 ± 0.09
3	8	5.0 × 10^7^	4.0	1.50	1.50	0.05	2.00	9	9.07 ± 0.38	0.60 ± 0.03
8	9	5.0 × 10^7^	6.0	0.02	0.02	0.05	2.00	0	26.26 ± 1.07	1.75 ± 0.07
9	10	5.0 × 10^7^	6.0	1.50	0.02	0.05	0.05	9	32.73 ± 2.12	2.18 ± 0.14
2	11	1.0 × 10^6^	6.0	1.50	0.02	2.00	2.00	9	18.54 ± 1.24	1.24 ± 0.08
4	12	1.0 × 10^6^	6.0	0.02	1.50	2.00	0.05	9	17.95 ± 0.95	1.20 ± 0.06

**Table 5:** Analysis of variance (ANOVA) from the Plackett–Burman design of ethanol production from PWH using *S. cerevisiae* HG1.1.

Source	Sum of square	df	Mean square	F value	*p* value Prob > F	Note
Model	l1264.96	7	180.71	34.46	0.0020	Significant
A-Cell concentration	341.76	1	341.76	65.17	0.0013	Significant
B-pH	840.01	1	840.01	160.17	0.0002	Significant
C- MnSO_4_.H_2_O	8.37	1	8.37	1.60	0.2752	
D- ZnSO_4_.7H_2_O	32.87	1	32.87	6.27	0.0665	
E- MgSO_4_.7H_2_O	0.63	1	0.63	0.12	0.7454	
F- KH_2_PO_4_	0.021	1	0.021	3.973E−3	0.9528	
G-Yeast extract	41.29	1	41.29	7.87	0.0485	Significant
Residual	20.98	4	5.24			
Cor Total	1285.93	11				
Std. Dev	2.29		R-Squared		0.9837	
C.V. %	16.28		Adj R-Squared		0.9551

In PWH with low total sugars (ca. 103 g/L), both inorganic nitrogen sources and other salts were unnecessary. However, the initial cell concentration strongly affects the ethanol production rate. Higher initial cell concentrations can promote the fermentation rate and ethanol production efficiency. Techaparin et al.^28^ reported that when the initial cell concentration increased from 1.0 × 10^7^ to 3.0 × 10^8^ cells/mL, the ethanol concentrations from sweet sorghum juice were raised from 64.79 to 84.32 g/L using *S. cerevisiae* KKU-VN8. Greater than ten times higher ethanol productivity from hydrolyzed sugarcane bagasse was achieved when the inoculum size of *S. cerevisiae* ITV-01 was increased from 0.2 to 10 g/L^47^.

Yeast growth and fermentation activity are affected directly by the pH of the fermentation medium. The enzymes involved in the yeast growth and ethanol production pathway may be inactivated at a low pH level^48^. Although *S. cerevisiae* can grow well at pH values between 4.0 and 6.0, the optimum pH for ethanol production is approximately 5.0–5.5. Singh and Bishnoi^49^ demonstrated that pH 5.5 was the optimum pH value from statistical optimization of ethanol production from pretreated wheat straw hydrolysate using *S. cerevisiae* MTCC 174. Izmirlioglu and Demirci^50^ also found that pH 5.5 was the optimum value for ethanol production from potato mash waste using *S. cerevisiae* ATCC 24,859, which yielded a 30.99 g/L ethanol concentration. This pH value was also the optimum condition for ethanol production using *K. marxianus* NIRE-K3 at 45 °C, providing a 93.2% yield efficiency and 0.48 g/g ethanol yield^44^.

Yeast extract is widely used as the primary organic nitrogen source in several ethanol fermentation processes. It has been recognized as having a highly positive effect on ethanol production^44,51^. In the present study, 4.95 g/L yeast extract was the optimum concentration for ethanol production from PWH by *S. cerevisiae* HG1.1. Different optimum concentrations of yeast extract for ethanol production have also been reported. For instance, Schnierda et al.^51^ demonstrated that 9.43 and 9.24 g/L ethanol were attained from molasses-based medium (20 g/L sugar) supplemented with 0.5 g/L total yeast assimilable nitrogen by *S. cerevisiae* EC1118 and *I. orientalis* Y1161, respectively. Yeast extract at 3.0 g/L was determined to be the optimum concentration for ethanol production by *S. cerevisiae* NP01 using a fermentation medium containing 280 g/L sucrose^52^.

In comparison, yeast extract at 9.0 g/L promoted ethanol production from sweet sorghum juice containing 270 g/L total sugars using *S. cerevisiae* NP01^53^. Yeast extract is essential for efficient ethanol fermentation, especially under very high gravity fermentation conditions and high-temperature fermentation processes, but the most challenging factor is the high cost. Therefore, many scientists have tried to replace other low-cost organic nitrogen sources, such as dried spent yeast, corn-steep liquor, poultry meal, and feather meal. They have also demonstrated their potential application in the production of ethanol and other biochemicals^32,52^.

### Optimization conditions for ethanol production by *S. cerevisiae* HG1.1 using CCD

The experimental design codes and actual values of the significant independent factors, including initial cell concentration (5.0 × 10^6^ to 1.0 × 10^8^ cells/mL), pH (4.0 to 6.5), and yeast extract (3.0 to 12.0 g/L), are shown in Table 6. The observed ethanol concentrations from the CCD with 20 experimental runs were 19.10–33.54 g/L, and the predicted ethanol concentrations were 19.49–33.78 g/L (Table 7). The ethanol productivities were 1.59–2.80 g/L.h. The quadratic polynomial regression model and a second-order polynomial equation to predict the final ethanol concentration (PE) as a function of the fermentation variables were established, and the prediction equation was as follows:

$$\begin{aligned} {\text{PE}}\left( {{\text{g}}/{\text{L}}} \right) = & {31}.{19} + {2}.{\text{32A}} + {2}.{\text{52B}} - {1}.{\text{14C}} - {1}.{\text{65AB}} \\ & \;{ + }0.{\text{32AC}} + {1}.{\text{42BC}} - 0.{\text{46A}}^{{2}} - {1}.{\text{67B}}^{{2}} - 0.{\text{21C}}^{{2}} \\ \end{aligned}$$

**Table 6:** Codes and actual values of the independent factors for CCD on ethanol production from PWH using *S. cerevisiae* HG1.1.

Codes	Factors	Units	Levels				
			− 1.68	− 1	0	+ 1	+ 1.68
A	Cell concentration	cells/mL	5.0 × 10^6^	2.4 × 10^7^	5.3 × 10^7^	8.1 × 10^7^	1.0 × 10^8^
B	pH		4.5	4.9	5.5	6.1	6.5
C	Yeast extract	g/L	3.00	4.82	7.50	10.18	12.00

**Table 7:** The central composite design matrix for ethanol production using *S. cerevisiae* HG1.1 at 40 °C.

Std	Run	Inoculum (cells/mL)	pH	Yeast extract (g/L)	Ethanol (g/L)		Productivity (g/L.h)
					Predicted	Observed	
3	1	2.4E+07	6.09	4.82	30.75	31.13 ± 1.55	2.59 ± 0.13
4	2	8.1E+07	6.09	4.82	31.45	31.96 ± 1.34	2.66 ± 0.11
17	3	5.3E+07	5.50	7.50	31.19	31.39 ± 1.71	2.62 ± 0.14
10	4	1.0E+08	5.50	7.50	33.78	33.54 ± 0.33	2.80 ± 0.03
1	5	2.4E+07	4.91	4.82	25.26	25.56 ± 0.71	2.13 ± 0.06
11	6	5.3E+07	4.50	7.50	22.22	22.23 ± 2.16	1.85 ± 0.19
13	7	5.3E+07	5.50	3.00	32.53	31.62 ± 1.19	2.64 ± 0.10
6	8	8.1E+07	4.91	10.18	28.05	27.79 ± 0.88	2.32 ± 0.07
12	9	5.3E+07	6.50	7.50	30.71	30.52 ± 1.17	2.54 ± 0.10
2	10	8.1E+07	4.91	4.82	32.54	32.97 ± 0.35	2.75 ± 0.03
14	11	5.3E+07	5.50	12.00	28.69	29.43 ± 1.46	2.45 ± 0.12
5	12	2.4E+07	4.91	10.18	19.49	19.10 ± 0.41	1.59 ± 0.03
7	13	2.4E+07	6.09	10.18	30.67	30.36 ± 1.63	2.53 ± 0.14
8	14	8.1E+07	6.09	10.18	32.65	32.47 ± 1.77	2.71 ± 0.15
15	15	5.3E+07	5.50	7.50	31.19	30.01 ± 0.64	2.50 ± 0.05
16	16	5.3E+07	5.50	7.50	31.19	32.10 ± 0.71	2.68 ± 0.06
20	17	5.3E+07	5.50	7.50	31.19	31.65 ± 1.56	2.64 ± 0.13
19	18	5.3E+07	5.50	7.50	31.19	31.11 ± 0.59	2.59 ± 0.05
9	19	5.0E+06	5.50	7.50	25.99	26.06 ± 0.82	2.17 ± 0.07
18	20	5.3E+07	5.50	7.50	31.99	30.94 ± 0.52	2.58 ± 0.04

The results revealed that the model was statistically significant (*p* value < 0.0001) (Table 8). The model was reliable because the *p* value of lack of fit was not statistically significant (*p* value > 0.005), the R-squared was 0.9808, and the adjusted R-squared was 0.9635, which was close to the R-squared. The standard deviation and coefficient values were only 0.71 and 2.40%, respectively. ANOVA also demonstrated that all these factors strongly affected ethanol production from PWH by *S. cerevisiae* HG1.1 at 40 °C. The *p* values of these significant factors were less than 0.0001.

**Table 8:** Analysis of variance (ANOVA) and results of regression analysis of the CCD on ethanol production from PWH using *S. cerevisiae* HG1.1.

Source	Sum of square	df	Mean square	F value	*p* value Prob > F	Note
Model	258.11	9	28.68	56.79	< 0.0001	Significant
A	73.21	1	73.21	144.97	< 0.0001	
B	86.86	1	86.86	172.01	< 0.0001	
C	17.78	1	17.78	35.21	0.0001	
AB	21.65	1	21.65	42.87	< 0.0001	
AC	0.82	1	0.82	1.62	0.2316	
BC	16.19	1	16.19	32.06	0.0002	
A^2^	3.07	1	3.07	6.09	0.0333	
B^2^	40.32	1	40.32	79.85	< 0.0001	
C^2^	0.61	1	0.61	1.21	0.2980	
Residual	5.05	10	0.50			
Lack of Fit	2.51	5	0.50	0.99	0.5052	Not significant
Pure Error	2.54	5	0.51			
Cor Total	263.16	19				
SD	0.71		R-Squared		0.9808	
C.V. %	2.40		Adj R-Squared		0.9635	

**Figure 2:**
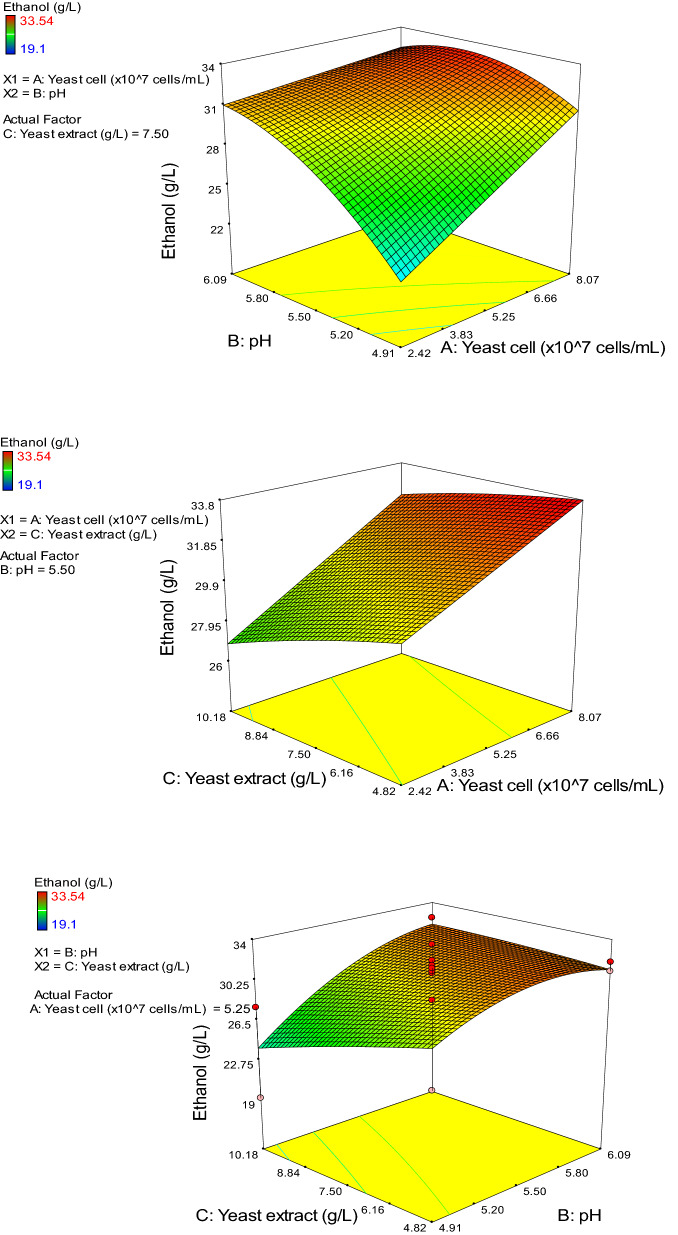
3-D response surface plots showing the effect of cell concentration (A), pH (B), and yeast extract (C) on ethanol concentration.

The 3-D response surfaces and contour plots for ethanol are presented in Fig. 2. The most fixed model was achieved when the yeast extract value was fixed at 7.50 g/L, and the cell concentration and pH levels were varied. The ethanol concentration was strongly affected by both cell concentration and pH. The maximum ethanol concentration of 33.54 g/L was achieved after 15 h of fermentation at the center pH value (pH 5.5) and cell concentration of 1.0 × 10^8^ cells/mL. The maximum ethanol productivity (2.80 g/L.h) was also attained. Based on the three-factor quadratic polynomial equation, the maximum predicted ethanol concentration was 33.67 g/L under the optimum conditions: cell concentration of 8.0 × 10^7^ cells/mL, pH of 5.4, and yeast extract concentration of 4.9 g/L.

Based on the result of the CCD experiment and the solution of the three-factor quadratic polynomial equation, three runs of experiments that gave high levels of ethanol were selected for a confirmatory experiment. A cell concentration of 8.0 × 10^7^ cells/mL, pH values in the range of 5.39–5.50, and yeast extract concentrations of 4.90–4.97 g/L were chosen for the confirmation test. The maximum ethanol concentration of 36.85 g/L, the productivity of 3.07 g/L.h, the ethanol yield of 0.48 g/g, corresponding to a yield efficiency of 93.61%, and sugar consumption of 74.81% were achieved under the optimum conditions, i.e., cell concentration of 8.0 × 10^7^ cells/mL, pH of 5.5, and yeast extract concentration of 4.95 g/L. The ethanol concentrations of these three confirmatory runs were not significantly different (36.07–36.85 g/L) (Table 9).

**Table 9:** The experimental design and results of verification experiments.

Run	Cell conc. (cells/mL)	pH	Yeast extract (g/L)	Ethanol (P_E_, g/L)		Qp (g/L.h)	Y_P/S_ (g/g)	E_y_ (%)	S_C_ (%)	t (h)
				Predicted	Observed					
1	8.0 × 10^7^	5.40	4.90	33.67	36.07 ± 0.92	3.01 ± 0.08	0.47 ± 0.02	92.20 ± 2.98	74.38 ± 2.91	12
2	8.0 × 10^7^	5.39	4.97	33.65	36.55 ± 0.46	3.05 ± 0.04	0.48 ± 0.01	93.03 ± 1.15	74.68 ± 1.78	12
3	8.0 × 10^7^	5.50	4.95	33.63	36.85 ± 0.72	3.07 ± 0.06	0.48 ± 0.01	93.61 ± 2.56	74.81 ± 0.72	12

P_E_: ethanol concentration (g/L); Q_p_: ethanol productivity (g/L.h); Y_p/s_: ethanol yield (g/g); E_y_: yield efficiency (%); S_c_: sugar consumption (%); and t: fermentation time (h).

**Figure 3:**
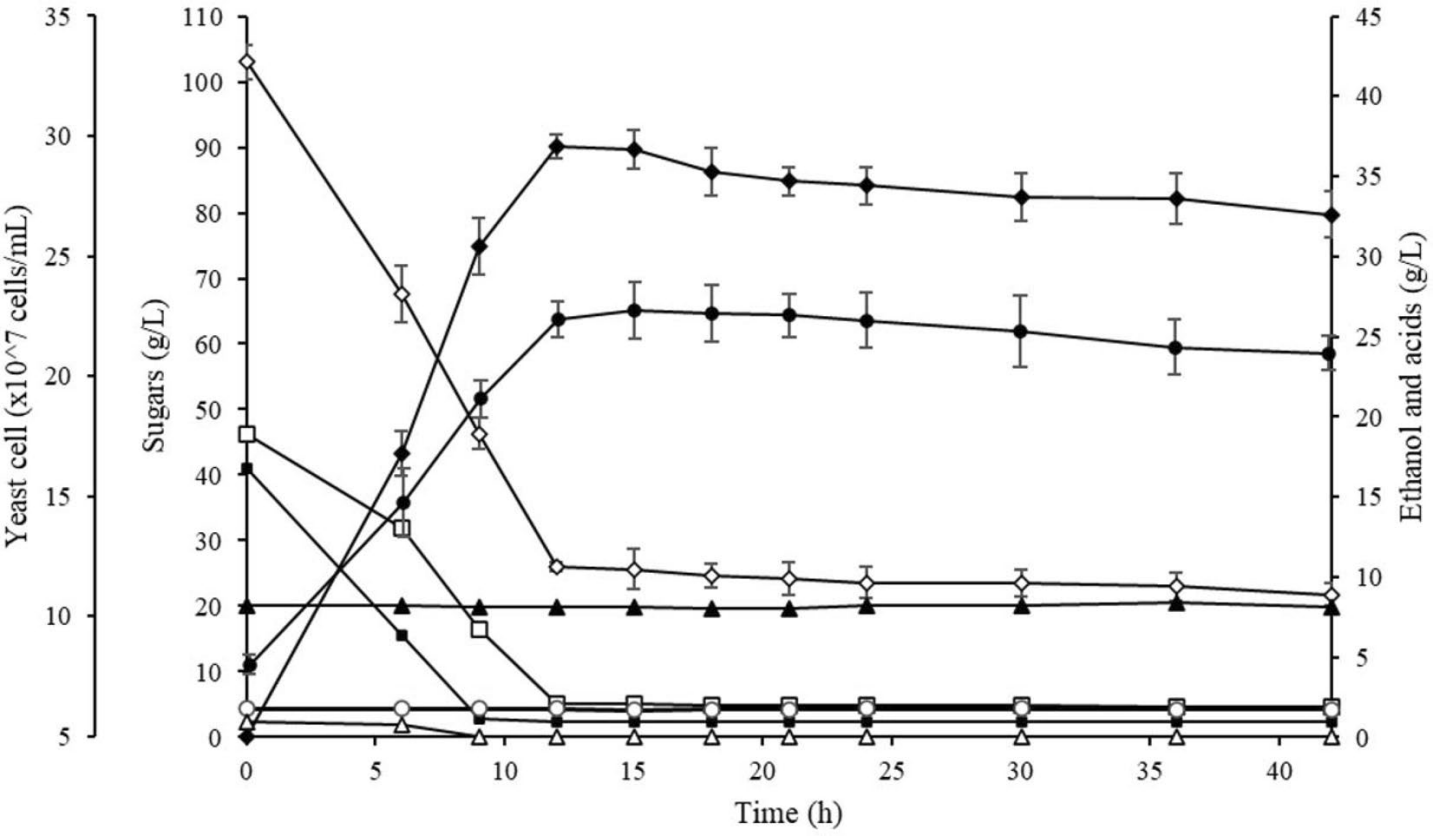
Time profile of ethanol production from pineapple waste hydrolysate using *S. cerevisiae* HG1.1 under optimum conditions. Symbols: filled black rhombus, ethanol; unfilled rhombus, total sugars; filled black square, glucose; unfilled square, fructose and xylose; unfilled circle, arabinose; unfilled triangle, formic acid; filled black triangle, acetic acid; filled black circle, yeast cells.

Figure 3 shows the time profile of ethanol production from PWH at 40 °C using *S. cerevisiae* HG1.1. The ethanol concentration quickly reached the maximal value (36.85 g/L) after 12 h of fermentation, corresponding to the dramatic decrease in total sugars (from 102.98 to 25.93 g/L). The ethanol content was slightly decreased after it reached the maximum concentration due to the oxidation of ethanol by yeast when the sugar in the fermentation medium was depleted. The remaining sugars, mostly C-5 sugars, such as xylose and arabinose, were almost unchanged since *S. cerevisiae* could not consume this type of sugar. The remaining total sugars in the fermented medium were 21.79 g/L. Although PWH contained some fermentation inhibitors, such as acetic acid (8.23 g/L), formic acid (0.96 g/L), and furfural (0.68 mg/L), the growth and fermentation activity of *S. cerevisiae* HG1.1 were not affected. The ethanol concentration, productivity, and yield efficiency achieved from PWH by *S. cerevisiae* HG1.1 at 40 °C were relatively high compared to several previous studies summarized in Table 10. This finding suggests that pineapple waste is a promising agricultural waste for second-generation bioethanol production.

**Table 10:** Comparative study on ethanol production from waste hydrolysate using *S. cerevisiae* and other yeast strains.

Strain	Substrate	T (°C)	S_0_ (g/L)	P_E_ (g/L)	Q_p_ (g/L.h)	E_y_ (%)	References
*S. cerevisiae* ATCC24859	Waste potato mash	30	137.0	32.90	3.59	92.16*	^50^
*S. cerevisiae* (Fleischmann’s yeast, USA)	Banana peels	37	48.0	28.20	2.30	74.51*	^54^
*S. cerevisiae* (Angel Yeast, China)	Pomelo peel waste	30	–	36.00	0.75	73.50	^55^
*S. cerevisiae* MTCC 174	Wheat straw	30	65.0	16.40	0.45	94.12*	^49^
*S. cerevisiae* KF-7	Rice straw	35	–	21.50	0.90*	77.30	^56^
*S. cerevisiae* TISTR 5048	Pineapple peel waste	30	82.1	27.33*	–	65.27	^38^
*K. marxianus* K21	Carrot pomace	42	–	37.00	0.88	42.80	^57^
*P. kudriavzevii* SI	Sugarcane bagasse	42	48.5	22.57	0.66	91.20	^58^
*I. orientalis* IPE100	Cornstalk	42	95.7	45.90	0.96	93.80	^59^
*S. cerevisiae* HG1.1	Pineapple waste	40	103.0	36.85	3.07	93.61	This study

T: temperature; S_0_: initial sugar concentration; P_E_: ethanol concentration; Q_p_: ethanol productivity; E_y_: yield efficiency; *: calculated by the authors (not given directly in the reference); –: not determined in the referenced study.

### RT–qPCR analysis of gene expression in thermotolerant *S. cerevisiae* HG1.1

Hundreds of genes possess different expressions in response to heat stress in yeast cells^60–62^. However, most previous gene expression studies were carried out using the synthetic medium. Only a few studies have shown the gene expression pattern using lignocellulosic materials as a feedstock. Thus, this study evaluated the expression of some groups of genes related to growth and ethanol production pathway, ethanol, oxidative and acetic acid stress, and DNA repair.

The expression levels of sixteen genes responsible for growth and ethanol stress (*ATP6*, *OLE1*, *ERG8*), oxidative stress (*GLR1*, *SOD1*), DNA repair (*RAD14*, *MRE11*, *POL4*), the pyruvate-to-TCA pathway (*PDA1*, *CIT1*, *LYS21*), the pyruvate-to-ethanol pathway (*PDC1*, *ADH1*, *ADH2*), and acetic acid stress (*ACS1*, *ALD2*) in *S. cerevisiae* HG 1.1 were successfully evaluated using RT–qPCR. As shown in Table 11, *ERG8*, *RAD14*, *ADH2*, and *ALD2* were up-regulated under a control growth condition (30 °C) and down-regulated under heat stress. *ATP6*, *GLR1*, *SOD1*, *CIT1*, *LYS21*, *ADH1*, and *ACS1* genes were highly expressed under heat shock at 40 °C for 30 min and markedly decreased under short- and long-term stress conditions. In contrast, the expression levels of *OLE1*, *MRE11*, *POL4*, *PDA1*, and *PDC1* were highly increased when yeast cells were shifted from 30 °C to 40 °C for 3 h (short-term stress) and then dramatically decreased when cells were shifted from 30 °C to 40 °C for 9 h (long-term stress). Under heat stress, the expression levels of the *PDC1* gene were more remarkable than that under a control condition. Relatively low expression levels of the *RAD14* gene responsible for DNA repair and the *ALD2* gene responsible for acetic acid stress were detected in yeast cells under long-term stress. It should be noted from the present study that genes involved in the same stress condition exhibited different expression patterns, suggesting their unique expression profile during high-temperature ethanol fermentation.

**Table 11:** Expression level of genes in the thermotolerant yeast *S. cerevisiae* HG1.1 during high-temperature ethanol fermentation using RT-qPCR.

Pathway or function involve	Gene	Product	Relative expression level			
			Unstressed (30 °C)	Heat shock	Short-term heat stress	Long-term heat stress
Response to growth and ethanol stress	*ATP6*	F1F0 ATP synthase subunit	1.48 ± 0.06	1.84 ± 0.10	1.68 ± 0.08	1.32 ± 0.06
	*OLE1*	Acyl-CoA desaturase 1	1.60 ± 0.10	1.74 ± 0.12	1.86 ± 0.04	1.48 ± 0.05
	*ERG8*	Phosphomevalonate kinase	1.63 ± 0.12	1.40 ± 0.06	1.25 ± 0.08	0.86 ± 0.02
DNA repair	*RAD14*	DNA excision repair protein RAD14	1.16 ± 0.03	0.91 ± 0.04	0.88 ± 0.06	0.06 ± 0.01
	*MRE11*	Double-strand break repair protein	0.82 ± 0.06	1.20 ± 0.05	1.26 ± 0.05	0.84 ± 0.02
	*POL4*	DNA polymerase IV	0.80 ± 0.01	1.12 ± 0.04	1.21 ± 0.04	0.78 ± 0.03
Oxidative stress	*GLR1*	Glutathione reductase	0.82 ± 0.04	0.96 ± 0.10	0.92 ± 0.06	0.66 ± 0.03
	*SOD1*	Superoxide dismutase [Cu–Zn]	0.94 ± 0.08	1.22 ± 0.04	1.16 ± 0.03	1.06 ± 0.06
Pyruvate-to-TCA pathway	*PDA1*	Pyruvate dehydrogenase	1.98 ± 0.06	2.28 ± 0.10	2.36 ± 0.12	1.97 ± 0.06
	*CIT1*	Citrate synthase	0.92 ± 0.05	1.30 ± 0.06	1.18 ± 0.04	1.06 ± 0.07
	*LYS21*	Homocitrate synthase	0.69 ± 0.05	1.07 ± 0.07	0.88 ± 0.05	0.52 ± 0.02
Pyruvate-to-ethanol pathway	*PDC1*	Pyruvate decarboxylase	1.16 ± 0.04	1.40 ± 0.05	1.58 ± 0.08	1.32 ± 0.05
	*ADH1*	Alcohol dehydrogenase 1	1.78 ± 0.08	2.08 ± 0.09	1.94 ± 0.06	1.79 ± 0.03
	*ADH2*	Alcohol dehydrogenase 2	1.13 ± 0.06	1.09 ± 0.06	0.87 ± 0.05	0.62 ± 0.04
Response to acetic acid stress	*ACS1*	Acetate-CoA ligase 1	0.70 ± 0.05	1.06 ± 0.05	0.96 ± 0.04	0.55 ± 0.03
	*ALD2*	Aldehyde dehydrogenase	0.98 ± 0.06	0.92 ± 0.04	0.76 ± 0.05	0.08 ± 0.01

*ATP6*, *OLE1*, and *ERG8* are essential genes responsible for yeast growth and ethanol stress^63^. In response to heat and ethanol stresses, more ATP is needed for several biosynthesis processes that produce critical components to protect microbial cells, such as trehalose, glycogen, unsaturated fatty acids and heat shock proteins^63^. As shown in the present study, the expression of *ATP6* was triggered by a heat shock condition, and its expression was slightly decreased under short- and long-term heat stresses. *ATP6* mitochondrially encoded subunit a of the F0 sector of mitochondrial F1F0 ATP synthase. It integrates into the F0F1-ATPase complex and completes the process to yield a functional ATPase^64^. Thus, a high level of *ATP6* expression in *S. cerevisiae* HG1.1 might be correlated with ATP production under heat shock conditions. In *S. cerevisiae* sun049T and *K. marxianus* DMKU 3–1042, the *ATP6* gene is also highly up-regulated during high-temperature ethanol production at 38°C^65^ and 45°C^60^, respectively.

*OLE1,* encoding a fatty acid desaturase, synthesizes monounsaturated fatty acids, such as palmitoleic acid and oleic acid, from saturated fatty acids, such as palmitic acid and stearic acid^66^. These unsaturated fatty acids and ergosterol maintain membrane fluidity as an adaptive response to the physiochemical interaction of both temperature and ethanol stresses^67^. It has been reported in *S. cerevisiae* that the overexpression of the *OLE1* gene enhanced acetic acid tolerance and other stresses, such as ethanol, H_2_O_2_, NaCl, benzoic acid, diamide, and menadione^68^. In this study, the *OLE1* gene of *S. cerevisiae* HG1.1 was highly expressed under heat shock and short-term heat stress, which was different from that of Qiu and Jiang^69^, who demonstrated that the *OLE1* gene of *S. cerevisiae* M1 was approximately 2.2–3.0-fold overexpressed at 30 °C under very high gravity ethanol production. The overexpression of the *OLE1* gene at 30 °C was also observed in *S. cerevisiae* K-9 under shaking and static sake fermentation^70^. Based on the present study, *OLE1* is a heat-shock responsive gene in *S. cerevisiae* HG1.1. The *ERG8* gene encodes phosphomevalonate kinase, which converts phosphomevalonate to diphosphomevalonate using ATP in ergosterol biosynthesis^71^. In the present study, the *ERG8* gene was down-regulated under heat stress conditions, which coincided with Rossignol et al.^72^, who pointed out that most of the genes encoding proteins involved in ergosterol biosynthesis in *S. cerevisiae* EC1118, including *ERG8*, were down-regulated at high-temperature fermentation. In *S. cerevisiae* YZ1 and YF3, low levels of *ERG8* expression were also observed under high-temperature conditions (42 °C), resulting in a reduction in ergosterol accumulation^73^.

DNA damage, including base disruption, base loss, and strand breaks, is not only induced by exposure to environmental agents, such as heat, UV rays, ROS, and oxidizing agents but also spontaneously generated during cellular metabolism^18^. *RAD14*, *MRE11* and *POL4* are common genes that encode proteins or enzymes involved in the DNA repair of yeast^74,75^. The expression of the *RAD14* gene in *S. cerevisiae HG1.1* was decreased under heat stress, similar to that reported by Boiteux and Jinks-Robertson^76^. The *RAD14* gene is recognized as a DNA damage binding factor for nucleotide excision repair in the UV-damaged DNA of *S. cerevisiae*. This gene is not induced by heat stress. The other two genes, i.e., *MRE11* and *POL4*, were up-regulated under heat shock and short-term heat stress conditions, and their expression was slightly reduced under long-term heat stress. The present results were similar to those of the *MRE11* and *POL4* genes in *K. marxianus* DMKU 3–1042, in which both genes were up-regulated under high-temperature stress^60^. It was proposed from the present study that *MRE11* and *POL4* are involved in the DNA repair of *S. cerevisiae* HG1.1 under heat stress conditions.

Oxidative stress, by increasing the accumulation of reactive oxygen species (ROS), such as superoxide anions, hydrogen peroxide, and hydroxyl radicals, has been shown to cause denaturation of macromolecules, such as DNA, RNA, proteins, and lipids, in yeast cells. Several genes, including *SOD1* and *GLR1*, are responsible for oxidative stress in yeasts. The *SOD1* gene encodes superoxide dismutase, while the *GLR1* gene encodes glutathione reductase. Both proteins have been shown to scavenge the superoxide anion radical and hydrogen peroxide, which can then be converted to water by the action of catalases or peroxidases^19^. The up-regulation of *SOD1* and *GLR1* genes under heat stress might correlate with high ROS accumulation. The overproduction of superoxide dismutase and glutathione reductase in *S. cerevisiae* HG1.1 might be needed to convert oxidative substrates such as superoxide anion radicals to hydrogen peroxide and finally to H_2_O. Overexpression of *SOD1* and *GLR1* has also been reported in *K. marxianus* DMKU 3–1042 and *S. cerevisiae* YZ1 and YF3 under heat stress^60,77^. In *S. cerevisiae* M1, up-regulation of the *SOD1* gene and down-regulation of the *GLR1* gene under high ethanol and high osmotic pressure have been reported^69^. Based on this information, the *SOD1* gene, but not *GLR1*, can be activated by heat, ethanol, and osmotic stresses, depending on the yeast species.

*PDA1* and *CIT1* are involved in the pyruvate-to-TCA pathway. These genes in *S. cerevisiae* HG1.1 were up-regulated under heat stress conditions, particularly under heat shock and short-term heat stress. Under long-term heat stress, the expression levels of both genes were slightly reduced. The expression of the *PDA1* gene of *S. cerevisiae* HG1.1 was similar to that of *S. cerevisiae* Y-50316^63^. However, they differed somewhat from those reported in *S. cerevisiae* M1, where the *PDA1* gene was down-regulated while *CIT1* was up-regulated under heat stress^69^. In *S. cerevisiae* Y-50316, the expression of the *PDA1* gene is activated not only by heat but also by ethanol stress^63^. A high expression level of the *CIT1* gene has also been reported in *S. cerevisiae* when cells are exposed to a high temperature of 35 °C for 10 min. The increasing expression level of *CIT1* increased the conversion of acetyl-CoA into the TCA pathway, leading to the accumulation of metabolic intermediates involved in the stress response^16^.

*LYS21* encodes homocitrate synthase, which functions to synthesize homocitrate from acetyl-CoA and oxoglutarate. Homocitrate is a precursor for the biosynthesis of L-lysine, which plays an essential protective role in response to oxidative stress induced by hydrogen peroxide in *S. cerevisiae*^78^. Furthermore, the homocitrate synthase enzyme is also associated with the mechanism of DNA repair in the nucleus^79^. In the present study, the expression of *LYS21* was enhanced under heat shock and short-term heat stress, and its expression slightly decreased after exposure to long-term heat stress. In *K. marxianus* DMKU 3–1042, the *LYS21* gene is also up-regulated under heat stress at 45°C^60^. Therefore, it was proposed from this finding that the *S. cerevisiae* HG1.1 The *LYS21* gene may be involved in DNA repair under heat stress.

In yeast cells, *PDC1*, *ADH1*, and *ADH2* are involved in a pyruvate-to-ethanol pathway. These genes are highly expressed in the stationary growth phase of *K. marxianus* DBKKU Y-102 under heat stress at 45°C^32^. Down-regulation of the *ADH2* gene has been reported in *S. cerevisiae* KKU-VN8 under heat stress at 40°C^34^. In *S. cerevisiae* Y-50316, the expression of *ADH1* and *ADH2* is also induced by ethanol stress^63^. In this study, *PDC1* and *ADH1*, but not *ADH2*, were up-regulated under heat stress at 40 °C, suggesting that heat stress could trigger the expression of *PDC1* and *ADH1* genes while suppressing the expression of *ADH2* in *S. cerevisiae* HG1.1 during high-temperature ethanol fermentation. A high ethanol concentration produced by *S. cerevisiae* HG1.1 at 40 °C might also be correlated with the overexpression of *PDC1* and *ADH1* genes.

Several genes, including *ACS1* (encoded acetate-CoA ligase) and *ALD2* (encoded aldehyde dehydrogenase), are responsible for acetic acid stress. The expression of these genes in *S. cerevisiae* HG1.1 under heat stress was investigated in this study. The results revealed that *ACS1* was up-regulated under heat shock and short-term heat stress, whereas *ALD2* was down-regulated under all stress conditions. The up-regulation of *ACS1* in *S. cerevisiae* HG1.1 under heat stress may lead to a high formation of acetic acid from acetyl-CoA but not from acetaldehyde because aldehyde dehydrogenase also utilizes NAD(P) + . The conversion of acetyl-CoA to acetic acid might generate more ATP, which can be used as an energy source for the biosynthesis of essential components or enzymes critical for yeast adaptation under heat stress. In *K. marxianus* DMKU 3–1042, *ACS1* and *ALD2* are up-regulated under heat stress at 45°C^60^, while they are highly expressed in *S. cerevisiae* M1 under normal growth condition (30 °C)^69^, suggesting that their expression profiles depend on the yeast species.

## Conclusion

The maximum ethanol concentration of 36.85 g/L, the productivity of 3.07 g/L.h, and yield efficiency of 93.61% were achieved after fermentation of PWH using *S. cerevisiae* HG1.1 at 40 °C under the optimum yeast inoculum concentration of 8.0 × 10^7^ cells/mL, pH of 5.5, and yeast extract concentration of 4.95 g/L. The expression of genes during high-temperature ethanol fermentation using RT–qPCR revealed that most of the genes, except *ERG8*, *RAD14*, *ADH2*, and *ALD2*, were up-regulated under heat stress conditions, particularly under heat shock and short-term heat stress. Interestingly, up-regulation of the *SOD1*, *PDA1, CIT1, PDC1,* and *ADH1* genes was observed under all stresses compared to a control treatment (unstress). Although the gene expression profiles were distinctive depending on the nature and characteristics of the yeasts, the thermotolerance acquisition and fermentation efficiency of *S. cerevisiae* HG1.1 during high-temperature ethanol fermentation correlated with genes responsible for growth and ethanol stress, oxidative stress, acetic acid stress, DNA repair, pyruvate-to-TCA, and pyruvate-to-ethanol pathway. These results provide useful information for further advanced research to explore the regulation of the genes to benefit this potential thermotolerant yeast for producing ethanol or other valuable bio-products under high-temperature fermentation conditions.

### Submission declaration and verification

Submission of an article implies that the work described has not been published previously in any form.

## Data Availability

The datasets used and/or analyzed during the current study are available from the corresponding author on reasonable request.

## References

[1] Ho DP, Ngo HH, Guo W (2014). A mini review on renewable sources for biofuel. Bioresour. Technol..

[2] Zabed H, Sahu JN, Suely A, Boyce AN, Faruq G (2017). Bioethanol production from renewable sources: Current perspectives and technological progress. Renew. Sustain. Energy Rev..

[3] Liu CM, Wu SY (2016). From biomass waste to biofuels and biomaterial building blocks. Renew. Energy..

[4] Moka S, Pande M, Rani M, Gakhar R, Sharma M, Rani J, Bhaskarwar AN (2014). Alternative fuels: An overview of current trends and scope for future. Renew. Sustain. Energy Rev..

[5] Naseeruddin S, Desai S, Venkateswar RL (2017). Ethanol production from lignocellulosic substrate Prosopis juliflora. Renew. Energy..

[6] Farrell AE, Plevin RJ, Turner BT, Jones AD, O'Hare M, Kammen DM (2006). Ethanol can contribute to energy and environmental goals. Science.

[7] Ketnawa S, Chaiwut P, Rawdkuen S (2012). Pineapple wastes: A potential source for bromelain extraction. Food Bioprod. Process..

[8] Choonut A, Saejong M, Sangkharak K (2014). The production of ethanol and hydrogen from pineapple peel by *Saccharomyces cerevisiae* and Enterobacter aerogenes. Energy Procedia..

[9] Aditiya HB, Chong WT, Mahlia TMI, Sebayang AH, Berawi MA, Nur H (2016). Second generation bioethanol potential from selected Malaysia's biodiversity biomasses: A review. Waste Manag..

[10] FAO regional office for Asia and the Pacific. FAO Statistical Yearbook 2014: Asia and the Pacific Food and Agriculture. (Bangkok, 2014).

[11] Sree KN, Sridhar M, Suresh K, Banat IM, Venkateswar RL (2000). Isolation of thermotolerant, osmotolerant, flocculating *Saccharomyces cerevisiae* for ethanol production. Bioresour. Technol..

[12] Yuangsaard N, Yongmanitchai W, Yamada M, Limtong S (2013). Selection and characterization of a newly isolated thermotolerant Pichia kudriavzevii strain for ethanol production at high temperature from cassava starch hydrolysate. Antonie Van Leeuwenhoek.

[13] Kwon YJ, Wang F, Liu CZ (2011). Deep-bed solid state fermentation of sweet sorghum stalk to ethanol by thermotolerant Issatchenkia orientalis IPE 100. Bioresour. Technol..

[14] Abdel-Banat BM, Hoshida H, Ano A, Nonklang S, Akada R (2010). High-temperature fermentation: How can processes for ethanol production at high temperatures become superior to the traditional process using mesophilic yeast?. Appl. Microbiol. Biotechnol..

[15] Limtong S, Sringiew C, Yongmanitchai W (2007). Production of fuel ethanol at high temperature from sugar cane juice by a newly isolated Kluyveromyces marxianus. Bioresour. Technol..

[16] Richter K, Haslbeck M, Buchner J (2010). The heat shock response: Life on the verge of death. Mol. Cell..

[17] Cadet J, Wagner JR (2013). DNA base damage by reactive oxygen species, oxidizing agents, and UV radiation. Cold Spring Harb. Perspect Biol..

[18] Tiwari S, Thakur R, Shankar J (2015). Role of heat-shock proteins in cellular function and in the biology of fungi. Biotechnol. Res. Int..

[19] Palma M, Guerreiro JF, Sá-Correia I (2018). Adaptive response and tolerance to acetic acid in *Saccharomyces cerevisiae* and Zygosaccharomyces bailii: A physiological genomics perspective. Front. Microbiol..

[20] Azhar SHM, Abdulla R, Jambo SA, Marbawi H, Gansau JA, Mohd FAA, Rodrigues KF (2017). Yeasts in sustainable bioethanol production: A review. Biochem. Biophys. Rep..

[21] Khatun MM, Yu X, Kondo A, Bai F, Zhao X (2017). Improved ethanol production at high temperature by consolidated bioprocessing using *Saccharomyces cerevisiae* strain engineered with artificial zinc finger protein. Bioresour. Technol..

[22] Thammasittirong SNR, Thirasaktana T, Thammasittirong A, Srisodsuk M (2013). Improvement of ethanol production by ethanol-tolerant Saccharomyces cerevisiae UVNR56. Springerplus.

[23] Phong HX, Klanrit P, Dung NTP, Yamada M, Thanonkeo P (2019). Isolation and characterization of thermotolerant yeasts for the production of second-generation bioethanol. Ann. Microbiol..

[24] Singh V, Haque S, Niwas R, Srivastava A, Pasupuleti M, Tripathi CKM (2016). Strategies for fermentation medium optimization: An in-depth review. Front. Microbiol..

[25] Antony J (2014). Design of Experiments for Engineers and Scientists.

[26] Khuri AI, Mukhopadhyay S (2010). Response surface methodology. Wiley Interdiscip. Rev. Comput. Stat..

[27] Nuanpeng S, Thanonkeo S, Yamada M, Thanonkeo P (2016). Ethanol production from sweet sorghum juice at high temperatures using a newly isolated thermotolerant yeast *Saccharomyces cerevisiae* DBKKU Y-53. Energies.

[28] Techaparin A, Thanonkeo P, Klanrit P (2017). High-temperature ethanol production using thermotolerant yeast newly isolated from Greater Mekong Subregion. Braz. J. Microbiol..

[29] Aruwajoye GS, Faloye FD, Kana EG (2020). Process optimisation of enzymatic saccharification of soaking assisted and thermal pretreated cassava peels waste for bioethanol. Waste Biomass Valor..

[30] Vogel KP, Pedersen JF, Masterson SD, Toy JJ, Fleet C, Lincoln C, Radison C, Fleet C, Lincoln C, Radison C (1999). Evaluation of a filter bag system forage analysis. Crop Sci..

[31] Rattanapoltee P, Kaewkannetra P (2014). Utilization of agricultural residues of pineapple peels and sugarcane bagasse as cost-saving raw materials in Scenedesmus acutus for lipid accumulation and biodiesel production. Appl. Biochem. Biotechnol..

[32] Charoensopharat K, Thanonkeo P, Thanonkeo S, Yamada M (2015). Ethanol production from Jerusalem artichoke tubers at high temperature by newly isolated thermotolerant inulin-utilizing yeast Kluyveromyces marxianus using consolidated bioprocessing. A. Van Leeuw. J. Microb..

[33] Koutinas M, Patsalou M, Stavrinou S, Vyrides I (2015). High temperature alcoholic fermentation of orange peel by the newly isolated thermotolerant Pichia kudriavzevii KVMP10. Lett. Appl. Microbiol..

[34] Techaparin A, Thanonkeo P, Klanrit P (2017). Gene expression profiles of the thermotolerant yeast *Saccharomyces cerevisiae* strain KKU-VN8 during high-temperature ethanol fermentation using sweet sorghum juice. Biotechnol. Lett..

[35] Zoecklein BW, Fugelsang KC, Gump BH, Nury FS, Zoecklein BW, Fugelsang KC, Gump BH, Nury FS (1995). Laboratory Procedures. Wine Analysis and Production.

[36] Mecozzi M (2005). Estimation of total carbohydrate amount in environmental samples by the phenol-sulphuric acid method assisted by multivariate calibration. Chemom. Intell. Lab. Syst..

[37] Laopaiboon L, Nuanpeng S, Srinophakun P, Klanrit P, Laopaiboon P (2009). Ethanol production from sweet sorghum juice using very high gravity technology: Effects of carbon and nitrogen supplementations. Bioresour. Technol..

[38] Niwaswong C, Chaiyamate P, Chotikosaikanon P, Ruangviriyachai C (2014). Simple and enhanced production of lignocellulosic ethanol by diluted acid hydrolysis process of pineapple peel (Ananas comosus) waste. African. J. Biotechnol..

[39] Sarkar N, Ghosh SK, Bannerjee S, Aikat K (2012). Bioethanol production from agricultural wastes: An overview. Renew. Energy..

[40] Saini JK, Saini R, Tewari L (2014). Lignocellulosic agriculture wastes as biomass feedstocks for second-generation bioethanol production: Concepts and recent developments. Biotech..

[41] Thomsen ST, Kádár Z, Schmidt JE (2014). Compositional analysis and projected biofuel potentials from common West African agricultural residues. Biomass Bioenergy..

[42] Deepshika K, Chauhan K (2014). Chemo-enzymatic conversion of biomass into bio-ethanol. J. Integr. Sci. Technol..

[43] Khongsay N, Lin YH, Laopaiboon P, Laopaiboon L (2014). Improvement of very-high-gravity ethanol fermentation from sweet sorghum juice by controlling fermentation redox potential. J. Taiwan Inst. Chem. Eng..

[44] Arora R, Behera S, Sharma NK, Kumar S (2017). Augmentation of ethanol production through statistically designed growth and fermentation medium using novel thermotolerant yeast isolates. Renew. Energy..

[45] Abd-Aziz S, Ang DC, Yusof HM, Karim MIA, Ariff AB, Uchiyama K, Shioya S (2001). Effect of C/N ratio and starch concentration on ethanol production from sago starch using recombinant yeast. World J. Microbiol. Biotechnol..

[46] Manikandan K, Viruthagiri T (2010). Optimization of C/N ratio of the medium and fermentation conditions of ethanol production from tapioca starch using co-culture of Aspergillus niger and *Saccharomyces cerevisiae*. Int. J. ChemTech Res..

[47] Gutiérrez-Rivera B, Ortiz-Muñiz B, Gómez-Rodríguez J, Cárdenas-Cágal A, Domínguez GJM, Aguilar-Uscanga MG (2015). Bioethanol production from hydrolyzed sugarcane bagasse supplemented with molasses “B” in a mixed yeast culture. Renew. Energy..

[48] Narendranath NV, Power R (2005). Relationship between pH and medium dissolved solids in terms of growth and metabolism of Lactobacilli and Saccharomyces cerevisiae during ethanol production. Appl. Environ. Microbiol..

[49] Singh A, Bishnoi NR (2013). Ethanol production from pretreated wheat straw hydrolyzate by *Saccharomyces cerevisiae* via sequential statistical optimization. Ind. Crops Prod..

[50] Izmirlioglu G, Demirci A (2012). Ethanol production from waste potato mash by using *Saccharomyces cerevisiae*. Appl. Sci..

[51] Schnierda T, Bauer FF, Divol B, van Rensburg E, Görgens JF (2014). Optimization of carbon and nitrogen medium components for biomass production using non-*Saccharomyces* wine yeasts. Lett. Appl. Microbiol..

[52] Chan-u-tit P, Laopaiboon L, Jaisil P, Laopaiboon P (2013). High level ethanol production by nitrogen and osmoprotectant supplementation under very high gravity fermentation conditions. Energies.

[53] Deesuth O, Laopaiboon P, Klanrit P, Laopaiboon L (2015). Improvement of ethanol production from sweet sorghum juice under high gravity and very high gravity conditions: Effects of nutrient supplementation and aeration. Ind. Crops Prod..

[54] Oberoi HS, Vadlani PV, Saida L, Bansal S, Hughes JD (2011). Ethanol production from banana peels using statistically optimized simultaneous saccharification and fermentation process. Waste Manag..

[55] Huang R, Cao M, Guo H, Qi W, Su R, He Z (2014). Enhanced ethanol production from pomelo peel waste by integrated hydrothermal treatment, multienzyme formulation, and fed-batch operation. J. Agric. Food Chem..

[56] Wang G, Tan L, Sun ZY, Gou ZX, Tang YQ, Kida K (2015). Production of bioethanol from rice straw by simultaneous saccharification and fermentation of whole pretreated slurry using *Saccharomyces cerevisiae* KF-7. Environ. Prog. Sustain. Energy..

[57] Yu CY, Jiang BH, Duan KJ (2013). Production of bioethanol from carrot pomace using the thermotolerant yeast Kluyveromyces marxianus. Energies.

[58] Yuan SF, Guo GL, Hwang WS (2017). Ethanol production from dilute-acid steam exploded lignocellulosic feedstocks using an isolated multistress-tolerant Pichia kudriavzevii strain. Microb. Biotechnol..

[59] Kwon YJ, Ma AZ, Li Q, Wang F, Zhuang GQ, Liu CZ (2011). Effect of lignocellulosic inhibitory compounds on growth and ethanol fermentation of newly-isolated thermotolerant Issatchenkia orientalis. Bioresour. Technol..

[60] Lertwattanasakul N, Kosaka T, Hosoyama A, Suzuki Y, Rodrussamee N, Matsutani M, Murata M, Fujimoto N, Suprayogi TK, Limtong S, Fujita N, Yamada M (2015). Genetic basis of the highly efficient yeast Kluyveromyces marxianus: Complete genome sequence and transcriptome analyses. Biotechnol. Biofuels..

[61] Varol D, Purutçuoglu V, Yilmaz R (2018). Transcriptomic analysis of the heat stress response for a commercial baker's yeast *Saccharomyces cerevisiae*. Genes Genom..

[62] Mühlhofer M, Berchtold E, Stratil CG, Csaba G, Kunold E, Bach NC, Sieber SA, Haslbeck M, Zimmer R, Buchner J (2019). The heat shock response in yeast maintains protein homeostasis by chaperoning and replenishing proteins. Cell Rep..

[63] Ma M, Liu LZ (2010). Quantitative transcription dynamic analysis reveals candidate genes and key regulators for ethanol tolerance in *Saccharomyces cerevisiae*. BMC Microbiol..

[64] García JJ, Ogilvie I, Robinson BH, Capaldi RA (2000). Structure, functioning, and assembly of the ATP synthase in cells from patients with the T8993G mitochondrial DNA mutation. J. Biol. Chem..

[65] Ismail KSK, Sakamoto T, Hatanaka H, Hasunuma T, Kondo A (2013). Gene expression cross-profiling in genetically modified industrial Saccharomyces cerevisiae strains during high-temperature ethanol production from xylose. J. Biotechnol..

[66] Kim HS, Kim NR, Choi W (2011). Total fatty acid content of the plasma membrane of Saccharomyces cerevisiae is more responsible for ethanol tolerance than the degree of unsaturation. Biotechnol. Lett..

[67] Vanegas JM, Contreras MF, Faller R, Longo ML (2012). Role of unsaturated lipid and ergosterol in ethanol tolerance of model yeast biomembranes. Biophys. J..

[68] Nasution O, Lee YM, Kim E, Lee Y, Kim W, Choi W (2017). Overexpression of OLE1 enhances stress tolerance and constitutively activates the MAPK HOG pathway in *Saccharomyces cerevisiae*. Biotechnol. Bioeng..

[69] Qiu Z, Jiang R (2017). Improving *Saccharomyces cerevisiae* ethanol production and tolerance via RNA polymerase II subunit Rpb7. Biotechnol. Biofuels..

[70] Shobayashi M, Ukena E, Fujii T, Iefuji H (2007). Genome-wide expression profile of sake brewing yeast under shaking and static conditions. Biosci. Biotechnol. Biochem..

[71] Chemler J, Yan Y, Koffas M (2006). Biosynthesis of isoprenoids, polyunsaturated fatty acids and flavonoids in *Saccharomyces cerevisiae*. Microb. Cell Fact..

[72] Rossignol T, Dulau L, Julien A, Blondin B (2003). Genome-wide monitoring of wine yeast gene expression during alcoholic fermentation. Yeast.

[73] Zhang M, Shi J, Jiang L (2015). Modulation of mitochondrial membrane integrity and ROS formation by high temperature in *Saccharomyces cerevisiae*. Electron. J. Biotechnol..

[74] Liu Y, Sung S, Kim Y, Li F, Gwon G, Jo A, Kim A, Kim T, Song O, Lee SE, Cho Y (2016). ATP-dependent DNA binding, unwinding, and resection by the Mre11/Rad50 complex. EMBO J..

[75] Galli A, Chan CY, Parfenova L, Cervelli T, Schiestl RH (2015). Requirement of POL3 and POL4 on non-homologous and microhomology-mediated end joining in rad50/xrs2 mutants of Saccharomyces cerevisiae. Mutagenesis.

[76] Boiteux S, Jinks-Robertson S (2013). DNA repair mechanisms and the bypass of DNA damage in Saccharomyces cerevisiae. Genetics.

[77] Landolfo S, Politi H, Angelozzi D, Mannazzu I (2008). ROS accumulation and oxidative damage to cell structures in *Saccharomyces cerevisiae* wine strains during fermentation of high-sugar-containing medium. Biochim. Biophys. Acta. Gen. Subj..

[78] O’Doherty PJ, Lyons V, Tun NM, Rogers PJ, Bailey TD, Wu MJ (2014). Transcriptomic and biochemical evidence for the role of lysine biosynthesis against linoleic acid hydroperoxide-induced stress in *Saccharomyces cerevisiae*. Free Radic. Res..

[79] Scott EM, Pillus L (2010). Homocitrate synthase connects amino acid metabolism to chromatin functions through Esa1 and DNA damage. Genes. Dev..

